# Short-term dynamics of causal information transfer in thalamocortical networks during natural inputs and microstimulation for somatosensory neuroprosthesis

**DOI:** 10.3389/fneng.2014.00036

**Published:** 2014-09-09

**Authors:** Mulugeta Semework, Marcello DiStasio

**Affiliations:** ^1^Department of Neuroscience, Columbia UniversityNew York, NY, USA; ^2^Biomedical Engineering Program, SUNY Downstate Medical Center and NYU PolytechnicBrooklyn, New York, NY, USA

**Keywords:** Linear Granger causality, somatosensory neuroprosthesis, brain-machine interface, sensory feedback, VPL

## Abstract

Recording the activity of large populations of neurons requires new methods to analyze and use the large volumes of time series data thus created. Fast and clear methods for finding functional connectivity are an important step toward the goal of understanding neural processing. This problem presents itself readily in somatosensory neuroprosthesis (SSNP) research, which uses microstimulation (MiSt) to activate neural tissue to mimic natural stimuli, and has the capacity to potentiate, depotentiate, or even destroy functional connections. As the aim of SSNP engineering is artificially creating neural responses that resemble those observed during natural inputs, a central goal is describing the influence of MiSt on activity structure among groups of neurons, and how this structure may be altered to affect perception or behavior. In this paper, we demonstrate the concept of Granger causality, combined with maximum likelihood methods, applied to neural signals recorded before, during, and after natural and electrical stimulation. We show how these analyses can be used to evaluate the changing interactions in the thalamocortical somatosensory system in response to repeated perturbation. Using LFPs recorded from the ventral posterolateral thalamus (VPL) and somatosensory cortex (S1) in anesthetized rats, we estimated pair-wise functional interactions between functional microdomains. The preliminary results demonstrate input-dependent modulations in the direction and strength of information flow during and after application of MiSt. Cortico-cortical interactions during cortical MiSt and baseline conditions showed the largest causal influence differences, while there was no statistically significant difference between pre- and post-stimulation baseline causal activities. These functional connectivity changes agree with physiologically accepted communication patterns through the network, and their particular parameters have implications for both rehabilitation and brain—machine interface SSNP applications.

## Introduction

Sensory feedback is essential for an animal to sample the environment and to control its own movements with speed and precision. It also represents an essential component of human motor function, affecting dexterity, locomotion, and the richness with which we experience our surroundings. We are therefore investigating how we may best influence the nervous system, particularly by generating signals akin to somatosensory percepts via microstimulation (MiSt) administered to two main input areas, the ventral posterolateral thalamus (VPL) and the somatosensory cortex.

This paper is based on prior rat work in which neural responses were recorded using microwire arrays implanted in the forepaw representation region of primary somatosensory cortex and thalamic subnuclei. By varying the amplitude, duration and the timing of MiSt current pulses, some aspects of the neuronal responses can be modulated, including the amplitude, duration and refractory period (McIntyre and Gunter, 1979; McIntyre et al., 1985, 2006; Romo et al., 1998, 2000; Grill and Kirsch, 2000; Salinas et al., 2000; Daly et al., 2012).

Many brain-machine interfaces aim to restore lost motor functions by channeling movement-related brain signals to end-effectors (such as a robotic hand or arm) bypassing compromised parts of the central nervous system (CNS), and their performance in novel tasks could possibly be greatly improved by providing the user with a real-time feedback about the external environment. As has been confirmed with behavioral experiments (Talwar et al., 2002; Fitzsimmons et al., 2007; London et al., 2008; Do et al., 2011, 2012; Semprini et al., 2012), MiSt has the potential to be used as sensory feedback for closed-loop brain-machine-interface (BMI) devices (Vato et al., 2012; Liao et al., 2013). However, the short- and long-term effects of such artificial input to the brain have not been fully described.

Human S1 electrical stimulation experiments have indicated that there is mostly a feeling of “tingling” instead of touch when MiSt is applied to the cortex or thalamus (Libet, 1964; Lenz and Dougherty, 1998; Kiss et al., 2003), although the source of this mismatch has not been explained. The process by which neural activity generates conscious percept is complex, but clearly relies heavily on shared processing and broadcasting by interconnected population of neurons. This calls for investigation of the presence or absence of modulations in the strength and direction of information flow within and between networks and brain regions involved in fast sensory discrimination tasks. A major goal for the field of sensory neurophysiology is to understand the rules by which pathways for neuronal information flow are selected during stimulus processing. It is therefore essential to carry out functional connectivity analysis during different stimulus presentations especially on stimulus driven networks such as the somatosensory system where an external input such as natural touch or MiSt produces a very dependable neuronal response pattern. This pattern is different from baseline activity and presumably has to modify something for it to appear and be interpreted as different from any other. Investigating connectivity strength and directionality makes it possible to get a glimpse of such a pattern and its modulation by external stimulus (Nykamp, 2009).

While decoding motor behavior from cortical neurons to control a prosthetic device can be relatively accurate, sending a very specific somatosensory signal to the brain using similar methods may not be achieved with the same success. The question then arises: what happens to the ongoing network activity, particularly directional influences, when we try to talk to the brain using direct microstimulation? One crucial unanswered question is how functional relationships between the activities of pairs or groups of neurons in the network would be affected during repeated application of different MiSt regimes. This is a very important question because changes in linear causal couplings could be very detrimental to how the brain interprets its environment. Various neural interactions are believed to be important for perceptual and cognitive functions, such as those that give rise to spatial acuity in tactile discrimination tasks by organizing different neocortical regions (Berk et al., 2006). One main target of SSNP, tactile discrimination, is affected by neural interactions with other sensory modalities from other brain regions, such as vision and visual imagery activities of the visual cortex (Zangaladze et al., 1999; Sathian and Zangaladze, 2001, 2002).

Our experimental paradigm involves mechanically stimulating touch receptors on the hands or arms of rats while recording the neural responses in the two aforementioned brain areas. We then use MiSt in one of the two brain regions and try to optimize it such that the somatosensory cortical responses are as similar as possible to those induced via natural touch. Previous results (Francis et al., 2008) recorded from the rat VPL indicate that there is a significant amount of proprioceptive information in the rostral (rVPL), focal cutaneous receptive fields in the middle (mVPL), and broad receptive fields in the caudal VPL (cVPL).

In this paper, we present our findings from an application of an auto-regressive (AR) model of Linear Granger Causality (LGC) to local field potential (LFP) signals recorded during and following MiSt and natural touch to identify directions of influence and dynamics of such interactions under different stimulus conditions. LGC has been widely used as a powerful tool to find causal relationships in time series (Granger, 1969; He et al., 2014) and has been used widely in the analysis of neuroscience data (Kaminski et al., 2001; Hesse et al., 2003; Brovelli et al., 2004; Chen et al., 2006; Hamilton et al., 2011; Kim et al., 2011; Dong et al., 2012; Huang et al., 2014).

We investigated the effect of MiSt on the natural and functional interaction between all pair-wise combinations of LFP signals recorded through electrode arrays placed in the VPL thalamus and S1 cortex. It is believed that probing possible and easily tractable changes in interactions due to MiSt, whether it is because of increases or reductions in within- and between-network connectivity, is a good starting point toward planning functional SSNP architectures.

## Materials and methods

### Subjects, surgeries, and experimental procedures

All surgeries and post-operative care were approved by State of New York University (SUNY) Downstate Medical Center Institutional Animal Care and Use Committee (IACUC), conforming to National Institutes of Health (NIH) Guidelines. Surgical and histological procedures have been presented elsewhere fields (Francis et al., 2008) and thus we simply reference our previous work here.

Three female Long–Evans rats (Charles River Laboratories, Wilmington, MA), weighing between 250 and 450 g were implanted with arrays of chronic electrodes in the ventral posterior lateral thalamus (VPL) and somatosensory cortex where neural responses to hand/finger skin touch were recorded. In short, a small craniotomy was made above the cortical regions of interest. The dura was removed and an array of 2 by 8 tungsten electrodes (500 and 250 micrometer row and electrode spacing respectively, Tucker-Davis Technologies, Alachua, FL) were inserted through the cortical tissue to a depth of 1 to 1.5 mm for S1, and 5–6 mm for the VPL. During the lowering processes of these electrode arrays, a Plexon MAP system (Plexon, Inc., Dallas, TX) was used to visualize local potential recordings. In addition, the voltage signals were converted to sound using an audio amplifier to monitor the neuronal spiking activity while we stimulated the contralateral finger/arm/hand with touch and physical manipulation to properly localize the target regions of interest.

Once the arrays were at the desired depth, Surgilube (Division of Atlanta, Inc., Melville, NY) was placed over the exposed cortical surface and dental acrylic was built up to cement-in the array connectors and close the exposed skull. The surgical area was cleaned at the end of the surgery and topical triple antibiotic gel (neomycin sulfate, polymyxin B sulfate, and bacitracin zinc) was applied to the surgical wound. After recovery from surgery (typically 1 week), receptive field mapping for S1 and VPL neurons was done by listening to neural responses through an audio amplifier while touching and manipulating different parts of the body. Neural signals were amplified and filtered (at 154 Hz–8.8 kHz) and digitized (at 40 kHz). LFPs were calculated from extracellular recordings by band-limiting the signal to 3.3–300 Hz. These recordings were done at 2 kHz which was sufficiently greater than the Nyquist frequency (600 Hz). Recording and stimulation were done under mild anesthesia as it was difficult to keep the hand and fingers still for the tactile input and to prevent the rats from activating touch receptors by laying a hand/finger on objects. MiSt was also done in the same condition for a fair comparison of neural activity. About 35% less pentobarbital anesthesia compared to what is used for surgery (32.5 mg/kg, while surgery dose is 50 mg/kg) was found to be a good dose to keep a healthy level of neural activity and perfect stillness of the digits.

During recording, the rats were stimulated (natural touch) by mechanically activating touch receptors such as those found on the surface of the hand. This was achieved by driving a mechanical stimulator at a given amplitude and frequency. A vibrating probe driven by the motor delivered a single contact to the rat skin surface (an area of about 1 mm^2^) lasting about a millisecond. We have found this approach can generate neural responses to mechanical touch in specific receptive fields although there were infrequent imperfections, such as delays in the motor activation and touch strength which can vary as a result of small limb movements but can hardly be quantified from the neural response which on average gives a typical pattern. To minimize the effects of such minor variations, stimulations were repeated 180 times (2 Hz for 1.5 min) and average responses were analyzed. Responses to individual stimuli were recorded and set aside for further causality analysis. Driver signal and actual mechanical stimulator motor movement delay measures were done periodically using EMG leads in place of skin receptors, and when occasionally deemed necessary, offline re-alignment of event times was done.

Natural stimulation was followed by microstimulation in the somatosensory cortex or VPL thalamus. We microstimulated using charge-balanced, biphasic constant currents in bipolar electrode arrangement. The biphasic stimulation pulse was always cathodic first, pulse width was 0.2 ms per phase, amplitude was set at 25 μA and frequency was 2 Hz. The initial parameters for this work were chosen based on the available literature and from previous results in the lab in monkeys and rats, which indicated microstimulation current ranges which evoked cortical activity that resembled what was observed during natural stimulations. Our microstimulation choices were based on the fact that the current density (ID) near the electrode tip: *ID* = *I*π*r*^2^ is dependent on the distance from the tip and amount of current injected (Stoney et al., 1968; Ranck, 1975; Tsytsarev et al., 2008). More neurons are recruited by smaller tips as they have greater ID, making them ideal for focused activation of a brain tissue (hence the term “microstimulation”). Accordingly, using the surface area of our stimulating electrode (TDT microwires, 1711 or 3927 μm^2^ for the commonly used 33 and 50 μm thick wires both cut at 60°, TDT personal communication), we found that our results agreed with the few similar studies. Pulse widths of 100–600 μs and amplitude of 8 μA (0.8–4.8 nC, the average psychophysical threshold of single pulses being 2.0 nC) in the rat S1 (barrel cortex) have been shown to cause behavioral responses (licking) (Butovas and Schwarz, 2007), a measure that has been described as being close the threshold for evoking short-latency action potentials in neurons near the stimulation electrode (Butovas and Schwarz, 2007). The lowest effective stimulus parameter to cause short-latency action potentials in neurons near the stimulation electrode (2.25 nC, i.e., 30 μA, at 75 μs, at 2 Hz) in our rat experiments, agrees with these findings.

Our rat cortical MiSt results also agree with most related studies which aim at establishing effective combinations by using behavioral measures. For instance, successful induction of behavioral responses from monkeys has been achieved by MiSt in S1 cortex (area 3a) using charge balanced, 200 μs wide, biphasic pulses with 30–50 μA amplitude and train duration of 200–500 ms (London et al., 2008).

To summarize, appropriate stimulus strength-duration parameters were established based on the aforementioned principles and results. The threshold single pulse current intensity range in observing cortical response well above baseline and comparable to activity recorded during presentation of natural stimuli was shown to be 5–10 nC/phase (25–50 μA at 200 μs pulse width). Before settling on these parameters, we considered microstimulation artifacts, which were not uniformly linear in the stimulus space, and refractory period.

### Data collection and pre-processing

We used Matlab (Mathworks, Natick, MA), Neuroexplorer (Nex Technologies, Littleton, MA) and Offline Sorter (Plexon, Inc.) to process and conduct our data analysis. In comparing the various stimulation conditions, MiSt and noisy channels were excluded from analysis.

The LFP signals here were used for the causal interaction analysis using the Granger method, which requires a stationary signal. The method assumes a signal that has an autoregressive part and a white noise component with zero mean and finite variance.

At the beginning of every day's experiment, baseline neural activity (no stimulation) of S1 cortex and VPL thalamus units is recorded while the rat is under anesthesia as described in Section Subjects, Surgeries and Experimental Procedures. At the end of natural and artificial stimulations (5–10 min later tested), after-stimulation baseline is recorded (again without stimulation, still under anesthesia). To make sure there were no expectancy-induced LFP activity modulations right before a given stimulus was applied, baseline mean and standard deviation calculations were done in the −400 to −15 ms (pre-stimulus) window. Four hundred milliseconds (400 ms) before the stimulus was selected because the longest MiSt induced LFP activity change lasted about 100 ms, and as the stimuli were applied at 2 Hz that was the length of window it took for the activity to return back to baseline. Upon inspection of the signals, there was no or little visible input expectation-caused ramping up or down of LFP activity, possibly owing to the poisson arrival time of the stimuli. As a cautionary measure however, a 15 ms window (the average length for short-latency natural touch responses) before the stimulus was excluded from baseline parameters analysis.

The average waveform made from all valid artifact-free snippets of all valid trials' time series were self-normalized [(*x*-pre-stimulus mean)/pre-stimulus standard deviation, *x* being LFP value at time *t*], and this windowed response trace was passed on to the next step, stationarity control. The difference of LFP values at consecutive time points was taken and used in subsequent LGC analysis, in order to minimize the effect of drifting baseline and thus maximize stationarity.

### Causality measurement theory

AR models are used to model stochastic processes whose present values are dependent on a weighted sum of previous values and a normally and randomly distributed (zero mean and finite variance) white noise error. We present here the theoretical background and validation of our method using simple models. Consider signals *x* and *y* whose observations at times *t* = 1, 2, …., *T* observations have zero mean and their prediction errors (*e_x_* and *e_y_*), which are dependent only on the autoregressive (AR) history of order *p*, as stated in Equations 1 and 2.

(1) $$y(t)=\sum_{k=1}^{p}a_{y}(k)y(t-k)+e_{y}(t)$$

(2) $$x(t)=\sum_{k=1}^{p}a_{x}(k)x(t-k)+e_{x}(t)$$

If the two signals are related to each other's past history, bivariate autoregressive models (3 and 4) that use the past *p*-values of both *x* and *y* time series, can improve the prediction of each other (Granger, 1969). Accordingly we have,

(3) $$y(t)\text{​}=\text{​}\sum_{k=1}^{p}a_{yx}(k)x(t-k)+\sum_{k=1}^{p}a_{yy}(k)y(t-k)+e_{yx}(t)$$

(4) $$x(t)\text{​}=\text{​}\sum_{k=1}^{p}a_{xx}(k)x(t-k)+\sum_{k=1}^{p}a_{xy}(k)y(t-k)+e_{xy}(t)$$

In which, the error terms (*e_yx_* and *e_xy_*) now include the variance of linear prediction errors incorporating both *x* and *y* time series. These models can be extended to an unlimited number of signals by expanding the interaction coefficient matrices *a* and *e*. The AR models are constructed from solving Yule-Walker equations, which generate coefficients that minimize forward prediction errors (Takigawa et al., 1996, 2000; Jing et al., 2001).

#### Autoregressive model order

This is one of the very important steps in finding truly meaningful relationships between signals. The selection of the autoregressive order must be done correctly as has been emphasized by others (Lindsey and Jones, 1998; Gourevitch et al., 2006). Since model orders for the given signals are not known a priori, Akaike information criterion (AIC) (Akaike, 1974) is used to find the model order that captures the most variance in the data (Takigawa et al., 1996, 2000; Bernasconi and Konig, 1999). The goal of optimization using AIC is to reduce the number of free parameters without sacrificing information.

To find the best fit, after our initial assessments with visual inspection of data and prediction fit, a fully automated iterative while loop was run to probe the time lag spaces. We computed the fits using 2–20 ms lags. We then employed a specific form of AIC, corrected Akaike information criterion (AICc) (Hurvich et al., 1998), which has a high penalty (a sample size limit that reduces over-fitting which can increase with model complexity) with small data sets (Posada and Crandall, 2001) and defined as:

(5) AICc=(T+p/T−p−2)+ln(RSS/T))

Where *RSS* is the residual sum of squares, or the likelihood where normal and independent distribution of model errors is assumed, “ *T* ” is the sample size and “*p*” is the model order. These computations were done individually for each possible pair of LFP signals giving us a mode order value (*p*) of 2 ms. Others have found this exact value when applying the same procedures (Gourevitch et al., 2006).

#### Causality measurement theory and test and validation

Linear direct effects can be measured for our signals *x* and *y* using their sole linear prediction errors and comparing them to the variance of prediction errors when one signal's future values are estimated by adding the history of another one. The ratios of prediction errors of one signal over when using the bivariate model gives a measure of accuracy and therefore causal influences.

First, just using signal *x*'s own history, its unbiased variance of prediction error (its residual sum of squares) is evaluated as:

(6) $$\sum_{x|x^{-}}=\frac{1}{T-p}\sum_{t=1}^{T}e_{x^{2}}\left(t\right)=\frac{RSS_{x|x^{-}}}{T-p}$$

We can do the same for the bivariate model where we include *y* to test if we can improve on predicting *x*.

(7) $$\sum_{x|y^{-}x^{-}}=\frac{1}{T-2p}\sum_{t=1}^{N}e_{yx^{2}}\left(t\right)=\frac{RSS_{x|y^{-}x^{-}}}{T-2p}$$

If *y* influences *x* in a direct way, the log ratio:

(8) $$LGC_{y\to x}=\ln\;\frac{\sum_{x|x^{-}}}{\sum_{x|y^{-}x^{-}}}$$

is greater than 1 as the prediction error variance is larger when only *x* history is used to estimate it, and Granger causality is inferred (Ashley et al., 1980). The strength of the drive is statistically evaluated using Fisher's test by assuming no *y* → *x* causality (*a_yx_*(*k*) = 0 for *k* = 1, …, *p*),

(9) $$F_{y\to x}^{LGC}=\frac{T-p}{p}\left(e^{LGC_{y\to x}}-\frac{T-2p}{T-p}\right)\text{with F}(p,N-2p)$$

To find an empirical baseline against which the measured causality values in the real data can be compared to, a highly simplified model of a network of causal interactions (a synthetic data causal network made up of fairly complex artificial signals) was constructed and tested (see Gourevitch et al., 2006 for similar methodologies). In both model (during code verification) and real data sets the first half of the time series' were used as reference data, and the remaining half as test data. The model data (a periodic signal) was cut into two in the middle (half-second oscillations each). In the case of the LFP signals (real data), these halves both came from the after-stimulus response window described before. In both the real LFP data and artificial test signals, once the right model parameters were found, one pair-member signal was time shifted (forward by *p* milliseconds) or a specified portion of the time series was randomized. Using this analysis, we found that as low as 5% randomization or time shifting the data by as little as 2 ms completely destroyed the temporal relationships that there were no more statistically significant LGCs detected in formerly linked pairs.

## Results

Using LFP activities before, during, and after both natural touch and microstimulation conditions, differences and similarities in the possible causal interaction within and between S1 and VPL were measured by LGC methods.

**Figure 1:**
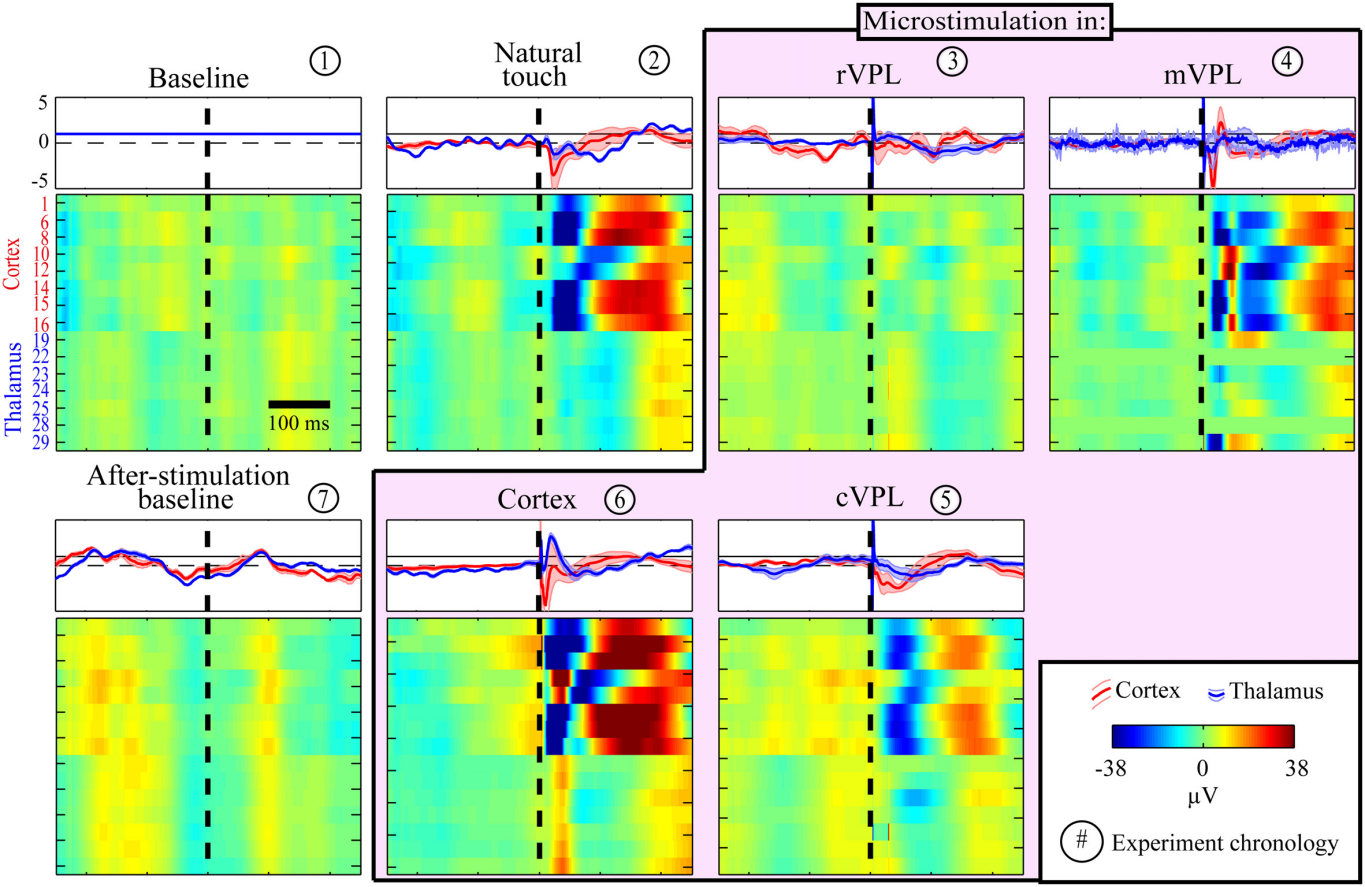
**Example session LFP response profiles to various (panel titles) stimulation and recording conditions**. Each row in each pixilated panel is the mean raw LFP activity for a given electrode and all responses are shown in the same amplitude scale (color bar in legend, in μV). These LFP peri-stimulus time histograms (PSTHs) for each electrode were calculated from a mean of 180 trials with stimulation delivered at time 0 (X-axis, black broken vertical line). The LFP pixel values were smoothed by averaging with a causal filter using a 50 ms sliding window. Y-axis in the first panel (baseline) enumerates the recording electrodes, top 8 rows being cortical and bottom 7 thalamic electrodes, none of which were noisy or used for microstimulation. A simple LFP responses similarity analyses between pre-stimulus baseline and other condition is plotted in the top inserts for each condition. This LFP modulation ratio is shown separately for cortical (red traces) and thalamic (blue) channels. Each trace represents a simple condition/baseline ratio, shown using the mean normalized response amplitude (thick line) and standard error of mean (shaded, *n* = 8 for cortex and *n* = 7 for thalamus). Note the alternating negative and positive deflections (almost always opposite for the post-stimulus period) of cortical and thalamic responses.

First, although our aim here is to study causal contributions but not to dissect and standardize the anatomical or temporal modulations of the LFP responses, we started with looking at the raw LFPs to get a perspective on simple similarities. A representative session LFP response indicating the generalized stimulus processing is shown in Figure 1. As expected from volume conduction, there is a general similarity between the LFP responses but there are differences such as negative deflections right after natural touch or microstimulation for cortical channels compared to thalamic ones. Note the common (roughly synchronized) activation of almost all thalamic LFPs during cVPL MiSt and in the after-microstimulation baseline. A simple ratio analysis, the mean response amplitude of cortical or thalamic channels due to a given condition over that of pre-stimulus baseline, is shown in the top inserts (rectangular panels) for each condition (square pixilated panels) in Figure 1. It shows a general post-stimulus modulation alternation between positive and negative deflections between cortical and thalamic channels.

**Figure 2:**
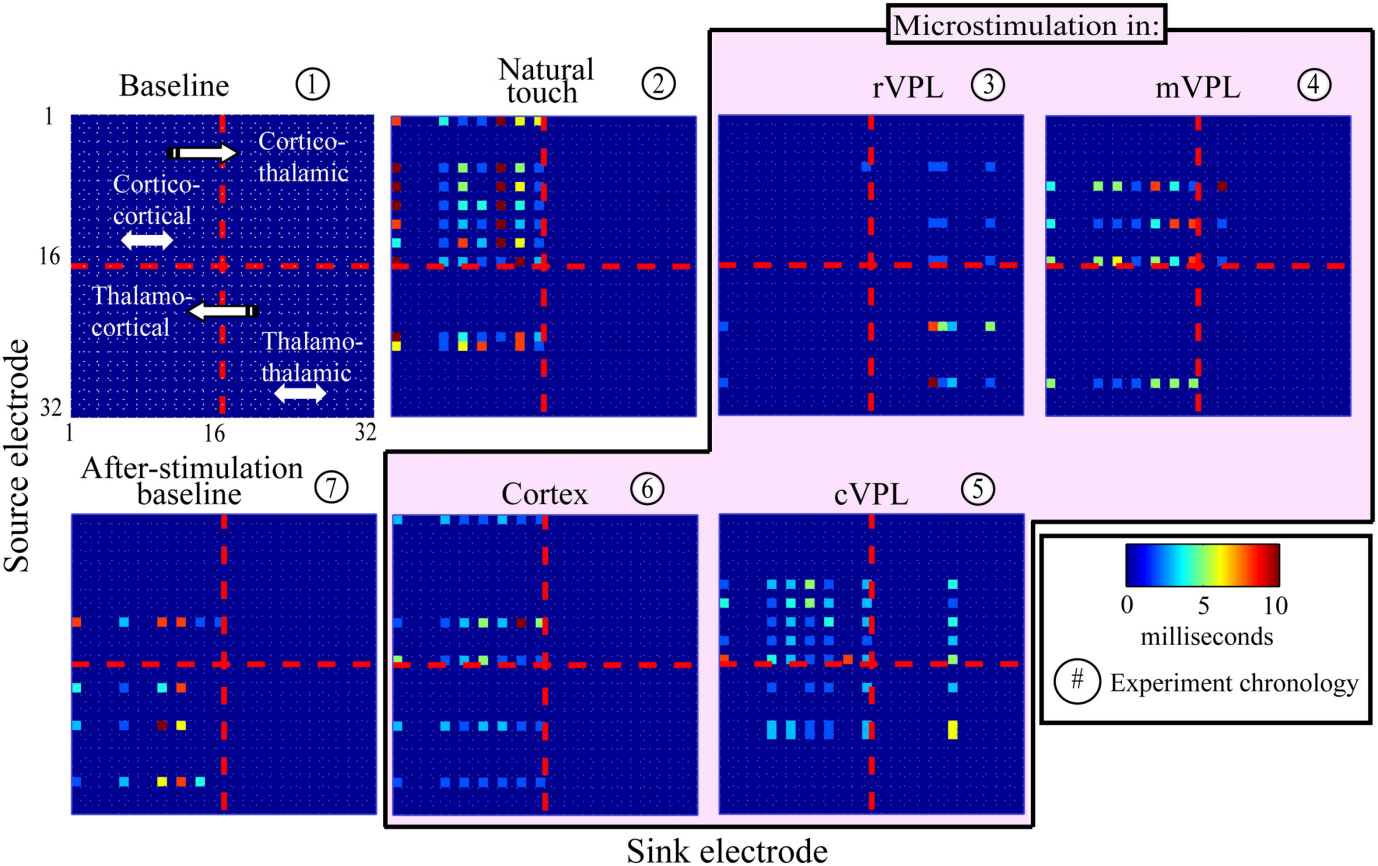
**An example of model temporal order-coded, statistically significant pair-wise causal interactions**. Data are from one recording session (mean of 180 trials; see Materials and Methods, Section Subjects, Surgeries, and Experimental Procedures). Electrode numbers 1–16 are located in primary somatosensory cortex, and numbers 17–32 are located in VPL thalamus in the anesthetized rat. Panel titles describe recording condition. Color bar shows model order (i.e., time offset in ms) that showed maximally significant causal links. Directions of causal influences are represented by the convention illustrated in panel 1. Channel pairs rarely had significant causalities at multiple model orders, but if they did, only the highest statistically significant (lowest *p*-value) one is displayed.

**Figure 3:**
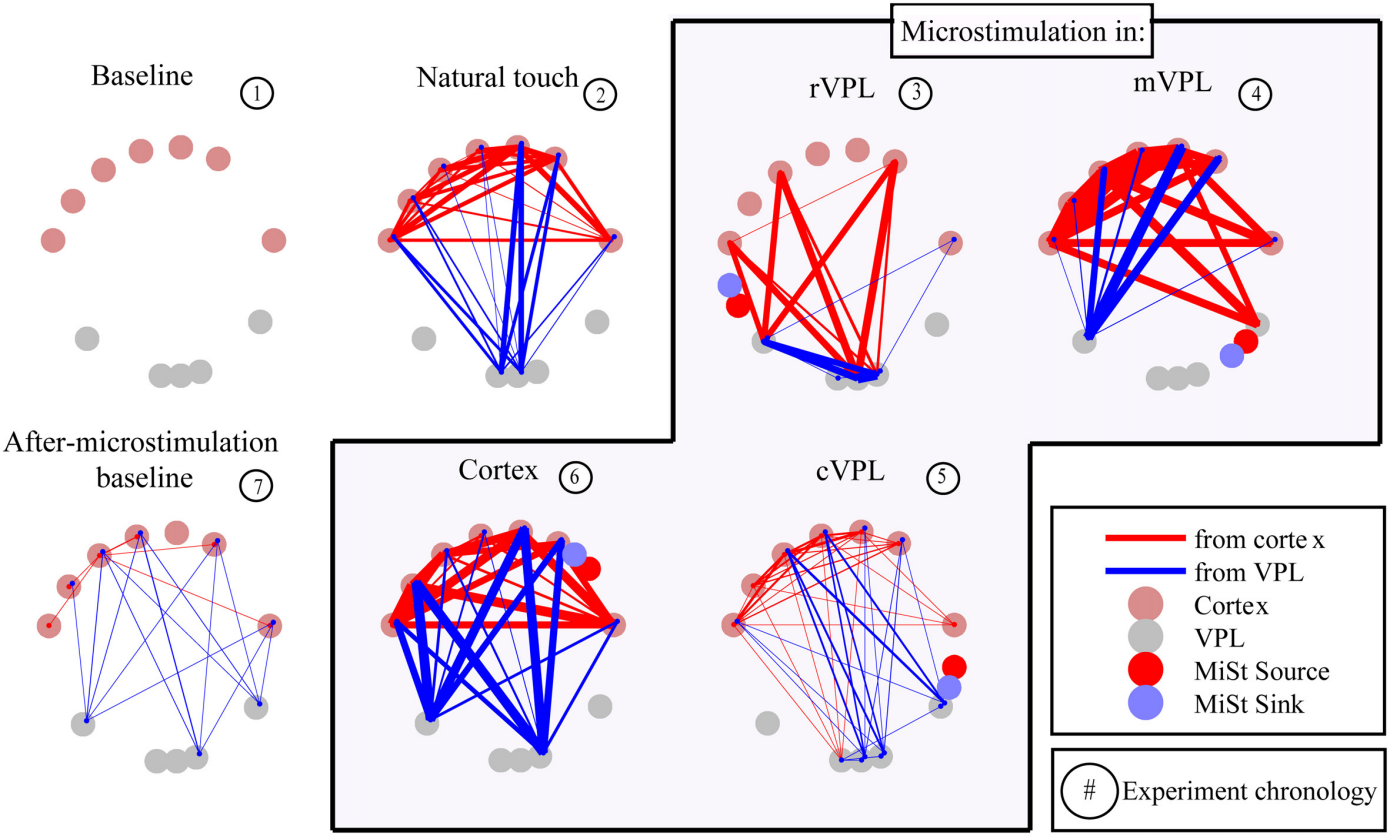
**Wiring diagram of significant Granger causalities for different directions of interactions and stimulation conditions between a set of 16 cortical (top tier) and 16 VPL (bottom) recording sites in the anesthetized rat (one session's data, mean of 180 trials)**. Some electrodes were excluded from analysis as they were used for microstimulation or there was noise. As shown in the legend, some electrodes were used for microstimulation while others were recorded from, both sets being in the same brain region. Wires designate the network of statistically significant causal information transfer within the set of the recording sites evaluated between each pair of electrodes. Thickness of the wires is proportional to the strength of the Granger causality (*F*^*LGC*^_*y* → *x*_, see Materials and Methods, Section Causality Measurement Theory and Test and Validation). Red lines are directional influences originating in cortex; blue lines are influences originating in VPL. Panel titles indicate recording condition. Microstimulation current source and sink (2 Hz, 25 μA, 200 μs, charge-balanced bipolar, biphasic, see Materials and Methods, Section Subjects, Surgeries, and Experimental Procedures) are represented by red and blue circles respectively.

The presence and absence of significant LGC between all pairs of LFP signals was first collected in a binary matrix where a “1” indicates a statistically significant (*p* < 0.001) causal influence from source to sink. Figure 2 presents an example (mean of 180 trials per condition) of the links discovered and it shows the binary map by color coding of the model order values that gave statistically significant causal influences. There are four quadrants in each panel (described in Figure 2 inset) that organize pair-wise influences based on electrode locations and the inferred direction of influence. These results are further depicted in Figure 3 which summarizes a single recording session's causal links in a wiring diagram (mean of 180 trials per condition) and shows static snapshots of the dynamics of causal influences by applying LGC analysis to a set of 16 cortical S1 and 16 VPL recording sites in one anesthetized rat (left hemisphere). Wires indicate the network of statistically significant causal information transfer within the set of recording sites. In addition to fluctuations in the overall trend of causal information flow under the different conditions, the intensity of the communication changes as shown by the line thickness in Figure 3.

These variations are observed in the gross amount of the information flow and the particulars (qualitative attributes) of the interactions. All reported results hold for all implants and sessions although there were minor variations which we assumed could possibly be explained by small deviations in electrode location and different natural thresholds. To verify if this was indeed true and to quantify the variability among the different subjects, we used the same simple procedures we employed for looking at GC probabilities (multicomparison *t* and *ks*) as well as non-parametric ones, Kruskal–Wallis and Wilcoxon–Mann–Whitney tests. All tests failed to show statistically significant variations (*p* > 0.01) between the subjects.

As a first approximation of communication trends, we looked at gross significant GC probabilities (summing all different directions of interactions for a given stimulation condition) which were calculated as percentages, i.e., electrodes found to have any statistically significant influence on their sinks divided by the total number of electrodes in the given brain region). These percentages, arranged in descending order are, cVPL MiSt (83%), mVPL MiSt (68%), CTX MiSt (67%), rVPL MiSt (46%), natural touch (44%), baseline (14%), and after-stimulation baseline (12%). Microstimulation in cVPL seems to cause the largest (bulk) information flow, probably owing to its diffuse cortical projections which also may be responsible for its broad receptive fields (Francis et al., 2008).

There is a huge influence within the cortex itself, from the cortex to the thalamus, and from the thalamus to the cortex. There is a significant amount of thalamo-thalamic interactions also, especially during VPL thalamic microstimulations. While there were differences between MiSt in the various VPL subnuclei and between the rats (not statistically significant as discussed below), these disparities disappear when the VPL is taken as a whole. As such, any MiSt in the VPL shows increased causal influence on the cortex.

**Figure 4:**
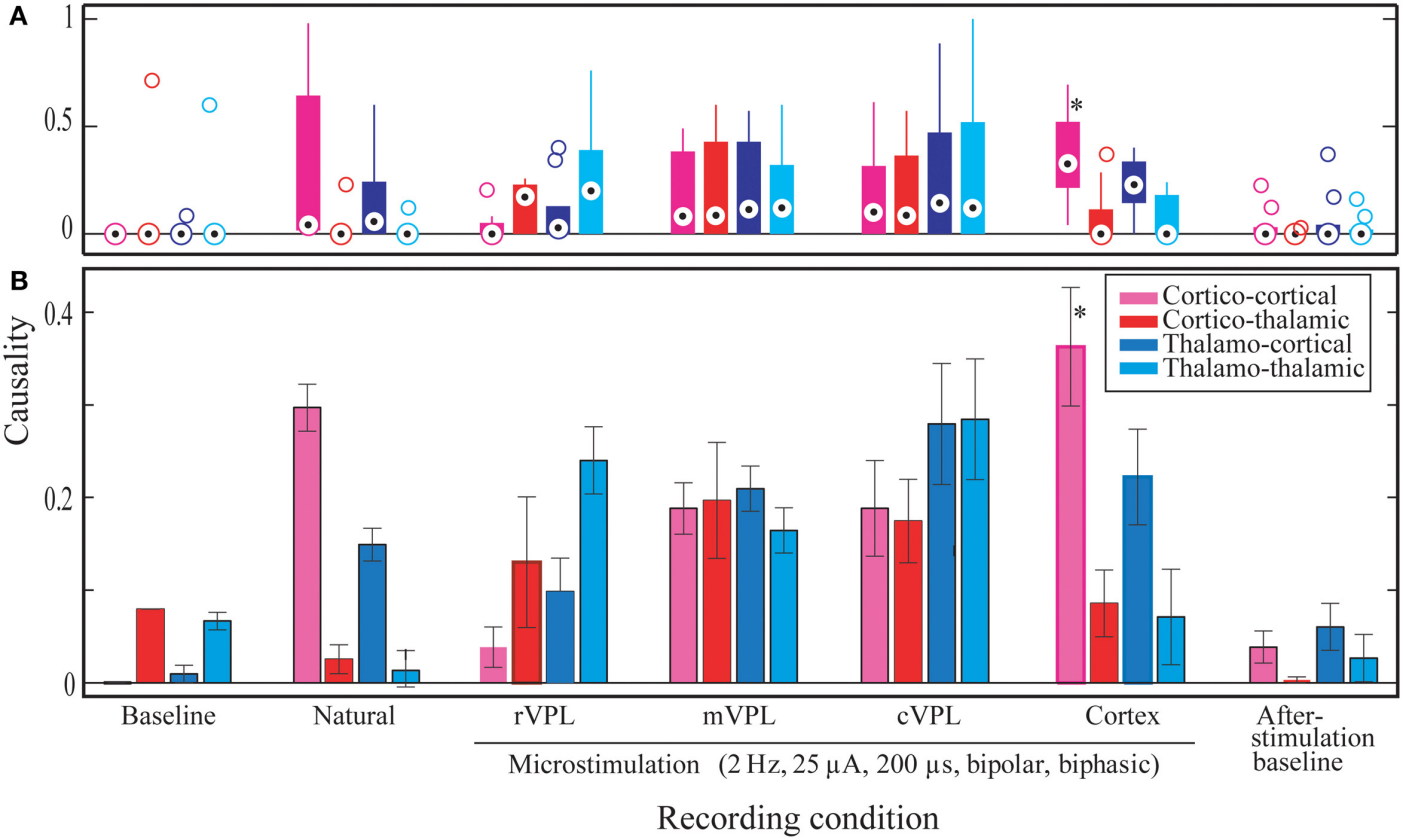
Significant Granger causality probabilities medians and means for different directions of interactions (x-axis) and stimulation conditions for all data from one hundred eighty (180) repetitions of each recording/stimulation condition per each of the three rats and 3 days of data each (*n* = 3 rats ^*^ 3 days ^*^ 180 trials = 1620 trials). Y-axis represents normalized ratios of electrodes found to have statistically significant influence on their sinks divided by the total number of electrodes in the given brain region. **(A)** The distribution of median significant Granger causality probabilities in the different conditions and directions (as specified in the x-axis below subplot **B**). Median values (session means) are shown by circles with central dots, the 25th and 75th percentiles by the edges of the box plots, and non-outlier data points covered by whisker extensions. Outliers are plotted as empty circles. **(B)** Mean comparisons. Whiskers correspond to the standard errors of mean (s.e.m.). Legend describes the color coding for the recording condition. Noisy and MiSt electrodes are excluded. All microstimulations: 2 Hz, 25 μA, 200 μs (charge-balanced bipolar, biphasic, see Materials and Methods, Section Subjects, Surgeries, and Experimental Procedures). Statistical outcomes are described in the Results section. Asterisk in the pink bar (Cortical microstimulation cortico-cortical LGC mean) indicates the most abundant statistically significant condition.

Statistical analyses were made to quantify the main differences between different conditions and directional influences (Figure 4). These statistical measures were applied to the comprehensive data from 180 repetitions of each recording/stimulation condition per each of the three rats and 3 days of data each (*n* = 3 rats ^*^ 3 days ^*^ 180 trials = 1620 trials). These grand-means and their standard errors of means (SEMs) are plotted in Subplot **(B)** in Figure 4.

As initial verification, the median number for the significant Granger causality probabilities was assessed in order to determine whether the data are symmetric or skewed (subplot **A** in Figure 4). This distribution analysis showed that, compared to baseline conditions, most have a positively skewed distribution (the median causal interaction level is raised). However, statistical analysis failed to classify these differences as significant when considering the same directions of interactions in the different stimulation/recording conditions. The only exception was cortico-cortical interaction during cortical microstimulation (CTX C-C) which showed a difference from pre-stimulus baseline (BL) C-C (median 0.33 vs. 0, *p* = 0.05).

To test whether a pair of conditions and directions have unknown variances but are independent random samples that come from normal distributions with equal means and variances, a paired *t*-test followed by a multicompare analysis of variance (ANOVA) statistical test was performed (Figure 4), and comparisons on the distributions of the values, i.e., on whether pairs were from the same continuous distribution, were made using a two-sample Kolmogorov–Smirnov (*k*s) test. Both tests, done at 0.01 *p*-value gave several differences. Table 1 presents the comparison between the significant pair comparison *p*-values.

**Table 1:** ***P*-values for mean LGC comparisons (done by pair-wise multi-comparisons) for the different directions of information flow and recording/stimulation locations and conditions**.

**Compared**	***p*-tt**	***p*-ks**	***p*-kw**	***p*-WMW**	**Abbreviations**
CTX C-C vs. BL C-C^*^	1E-04	5E-05	0.0005	0.0004	tt: *t*-test
CTX C-C vs. BL2 C-T	0.0001	5E-05	0.0002	4.11E-05	ks: Kolmogorov–Smirnov test (“D” statistic)
CTX C-C vs. BL T-C	0.0001	0.0005	0.0017	0.0009	kw: Kruskal–Wallis test
CTX C-C vs. NATURAL T-T	0.0002	0.0005	0.0003	8E-05	WMW: Wilcoxon–Mann–Whitney test
CTX T-C vs. BL C-C	0.0002	0.0005	0.0005	0.0004	CTX: cortical microstimulation
CTX T-C vs. BL2 C-T	0.0002	0.0005	0.0008	0.0004	BL: baseline;
CTX C-C vs. BL2 T-T	0.0003	0.0005	0.0005	0.0002	BL2: after-stimulation baseline;
CTX C-C vs. NATURAL C-T	0.0004	0.0005	0.0005	0.0002	C-C: cortico-cortical;
CTX T-C vs. BL T-C^*^	0.0004	0.0035	0.0011	0.0008	C-T: cortico-thalamic;
CTX C-C vs. rVPL C-C^*^	0.0005	0.0035	0.0010	0.0003	T-C: thalamo-cortical;
CTX T-C vs. NATURAL T-T	0.0005	0.0035	0.0011	0.0008	T-T: thalamo-thalamic;
CTX C-C vs. BL2 C-C^*^	0.0006	0.0035	0.0006	0.0002	rVPL: rostral subneucleus of the VPL thalamus
CTX T-C vs. BL2 T-T	0.0013	0.0035	0.0023	0.0013	
CTX T-C vs. NATURAL C-T	0.0018	0.0035	0.0029	0.0019	
CTX C-C vs. BL2 T-C	0.0021	0.0035	0.0017	0.0009	
CTX C-C vs. BL T-T	0.0076	0.0005	0.0020	0.0010	

*Note that p-values less than 0.01 indicate significant differences between the pair of compared means (all combinations are significant, column 1, p-tt ascending order). Statistical differences for the same direction of flow are indicated by asterisks*.

As shown in Table 1, there were several more significant differences which are mainly characterized by being limited to cortical microstimulation and baseline or natural stimulation conditions. We considered all directions and pairs to investigate what changes in a given condition and how significant are those changes are. Such measures are important to answer some questions such as: Are T-C interactions different from C-T in general? Under what conditions is one greater than the other? However our comments here largely focus on the same directions of interaction (highlighted white in Column 1 of Table 1).

Size wise, CTX C-C is the largest causality interaction and it is followed by natural touch cortico-cortical (natural C-C) communication. The two showed no statistical difference. The largest differences found (causal influence increase) was between CTX C-C and both baseline conditions (ttest2 *p* < 0.0001, and kstest2 *p* ≤ 0.00005). The relative amount of respective links for the four directions of interactions is visually similar between cortical microstimulation and natural touch conditions. Subplot **(B)** in Figure 4 shows that the different directions of interactions in the two conditions are comparable despite the fact they are all exaggerated in cortical microstimulation.

For pre-stimulation baseline conditions, the two within-brain region interactions (C-C and T-T) are different from all information flow directions during cortical microstimulation. For after-stimulation baseline, these differences (i.e., T-T and C-C of after-stimulation baseline) are true only when compared to CTX C-C (i.e., not different from the other directions during cortical microstimulation).

Baseline C-T causality didn't show statistically significant difference from that during cortical microstimulation while there was a difference in the after-stimulation baseline. Second, both after/before stimulation baselines are also different from cortical microstimulation conditions. Virtually all directions of causality interactions in the two baseline conditions are represented in these differences.

It is imperative to note one important exception here. While it is true that cortical microstimulation vs. baseline and natural stimulation conditions dominate the statistical findings, microstimulation in the rostral subnucleus of the VPL thalamus also different form CTX C-C (rVPL C-C, ttest2 *p* = 0.0005, and kstest2 *p* = 0.0035). The other directions of interactions when microstimulation in rVPL, and all of the m/cVPL conditions and directions were not significantly different from the rest of the recorded conditions.

Most of the statistically significant difference occurs between CTX C-C and both baseline conditions, quite understandably because of lack of significant interactions in the baseline conditions non-normal distributions there. Figure 4A shows the numerous zero-means (session means plotted) and skewed medians for graphic comparison purposes. As the goal here was not only to compare baselines to themselves and to different conditions but also the various stimulation conditions with each other, the evaluation of statistically significant differences is done in multi-comparison mode to isolate the pair-wise differences, as opposed to doing a simple in-group *t*-test. Considering a few outliers and making no assumptions about the data, we used nonparametric procedures. A multiple comparison test using Kruskal–Wallis structures gave similar conclusions as a simple pair-wise *t*-test but for some pairs it showed to be less-sensitive when compared to ANOVAs (0.0005 vs. 0.0009 for rVPL C-C vs. CTX C-C), indicating normal distribution. Understandably, this was not true when looking at the zero-means from baseline conditions and skewed medians were tested against CTX MiSt. Assuming non-normal distributions, another nonparametric test, Wilcoxon–Mann–Whitney test also was applied and it also found CTX C-C to be different from the baseline conditions and rVPL C-C (CTX C-C vs. rVPL C-C exact *p* = 0.0003, normal approx. *p* = 0.0011).

There was an exaggerated (overall) causal activity when microstimulating in the medial and caudal VPL thalamus subnuclei. For instance, cortico-thalamic causal interactions are highest during VPL thalamus microstimulation and in the immediate (5–10 min tested). Post-stimulation time thalamo-thalamic influences are highest also in this time window. However, these increases were not statistically different from any other condition. We believe the tested time-window is adequate as a starting position since a related work has shown that even a 30 min long microstimulation, although it causes functional changes, doesn't lead to long-term functional effects (Song et al., 2013).

One very important observation on this data is that there is no statistically significant difference between pre- and post-stimulation baseline causal activities.

## Discussion

Previous results from our monkey experiments have hinted that, compared to the cortex, MiSt in the ventral posterolateral nucleus of the thalamus (VPL) may produce cortical neural responses more closely resembling those recorded during natural touch. In the experiments presented here, baseline S1 and VPL activity was recorded, followed by natural stimulation (skin receptor activation) using a vibrotactile stimulator. This was followed by single-channel direct MiSt in the two brain regions using pulse width of 0.2 ms, amplitude of 25 μA, and frequency of 2 Hz (all charge-balanced, biphasic direct current in a bipolar electrode arrangement). Directional causal influences measures were then applied to the recorded LFP cortical and thalamic activities to determine differences and similarities and to establish working boundaries.

As expected, and demonstrated by the results presented here, network activity is dynamic and dependent on stimulus conditions. Causal interactions between and within S1 and VPL show short-term and reversible direction and strength modulations. Before implications and possible causes are explained, here we will briefly summarize the main causality measure results which in three important points. First, there are no statistical differences between pre- and post-stimulation conditions; second, cortical microstimulation has the largest and most abundant difference from baseline and natural stimulation conditions; and third, an increase or decrease in the grand-total of information does not directly translate into statistically significant causality changes.

Our results support the proposition that VPL thalamus has the potential for use as a microstimulation target for artificial somatosensory feedback. This is mainly because the preliminary data shows no statistically observable difference between natural stimulation and VPL microstimulation conditions. This information should be evaluated in terms of another expected but absent statistical difference, the one between microstimulation conditions (when MiSt is applied to either the cortex or VPL). This reality has not escaped our attention and we believe it may have to do with two facts. One, there was relatively little variability in our natural stimulation conditions compared to what is encountered during natural movements and exploration. Two, there is a broad increase in causal information flow during VPL microstimulation, which may overwhelm possible differences created in cortical microstimulation. As such, the largest (total) information flow happens during cVPL microstimulation which may be due to a gross activation of the VPL thalamus and the cortex, probably owing to its diffuse cortical projections which also may be responsible for its broad receptive fields (Francis et al., 2008). Even the only exception, rVPL microstimulation (rVPL C-C) being statistically different from cortical microstimulation (CTX C-C) may speak to the quality difference in the type of sensory information located in the two brain regions. Our cortical microstimulation was aimed at S1 cortex targeting touch sensation, while rVPL is implicated in proprioception (Francis et al., 2008).

As can be seen in the figures, there is a strong functional influence inside the cortex and from the cortex to the thalamus which may be explained by the fact that there is a strong cortico-thalamic feedback projection that modulates sensory information (Li and Ebner, 2007).

These results agree with three very important previously established facts: (1) there is a strong cortico-thalamic feedback projection that modulates sensory information (Li and Ebner, 2007); (2) there is a stronger neuronal interaction in the cortex than in the thalamus in the somatosensory pathway (Kim et al., 2003); and (3) the cortex drives thalamic relay neurons (Sherman and Guilery, 2006) which ultimately affects their sampling of natural peripheral inputs. Accordingly, our graphical and statistical evaluation of the linked LFP signals agrees with what can be expected from known physiological and anatomical relationships.

As we could sample only a small subset of cortical and thalamic neurons which have functional connectivities, the ones we recorded from cannot be assumed to tell the whole story. Most of our sampled cortical units are those that had short latency evoked responses (SLERUs) which, on average, responded within 5–10 ms of the natural (touch) input. There were a few with long latency evoked responses (LLERUs, responded within 15–20 ms). A neural population made up of both such units have been observed before in forepaw area of the rat S1 cortex (Shin et al., 1993, 1994). As the thalamus has more modulators (units that affect the way a signal is transmitted without changing its functional characteristics) in it been described as majorly concerned with sending messages from one cortical area to the other. This makes it a relay for transmitting information to the cerebral cortex along with functionally parallel driver pathways (Sherman and Guilery, 2006).

The short and long latency rat S1 cortical responses to natural touch have been described as representing transmission through different ascending somatosensory pathways (Lund and Webster, 1967; Chapin et al., 1986). LEURs were spared and SEURs were lost when contralateral cineaste nucleus was lessoned. This suggested SEURs are derived from transmission through the dorsal column-lemniscal system and LEURs from extralemniscal systems with possible secondary telencephalic processing of the SEURs. The higher neuronal interaction in the cortex may be related to this secondary processing. Another reason could be because the cortex extracts higher features of the sensory stimuli (Kim et al., 2003).

Some of these possibilities may explain a few of our observations that seem to be counterintuitive at first glance, for instance, the functional cortico-thalamic influence under baseline recording conditions. We believe this particular effect could be because the brain is normally active as the various areas and networks periodically poll each other and other non-stimulus related communication is always playing in the background. This may be a residual of an active sensing mechanism (Schroeder et al., 2010), which we will further investigate by having the animals in our experimental setting where they are alert and possibly attentively waiting for external inputs. It would be interesting to find which oscillation bands are represented under the different conditions. For now, we did not make an effort to use LGC to find band-based linkage as it may give misleading results in the case of causality on a spectral band (Gourevitch et al., 2006).

In conclusion, the lack of statistical differences between pre- and post-stimulus baseline causal interactions suggests that microstimulation and natural touch do not appear to permanently change directional influences in network activity in the time frame we recorded and analyzed the data (5–10 min post-stimulus tested). Moreover, the fact that 5–10 min after all stimulation conditions are done, there are no statistical differences between the within-brain-region interactions vs. out-of-area ones, indicates the after-effects of the stimulation conditions are quickly confined to the given brain region. The data presented here agree with related work (Song et al., 2013) showing these effects are not long-lasting.

There are several issues that have to be considered to further our understanding of the somatosensory system and neuroprosthetic interventions. One of the most important ones has to do with what happens to sensory perception during movement. In order to be able to isolate the effects of MiSt on sensory perceptions in the fine touch (lemniscal) pathway, one has to consider gating effects from the dorsal column nuclei which have long been shown to be modulated by arousal level, especially during movement (Ghez and Pisa, 1972). This is a very critical issue as somatosensory neuroprosthetic applications of MiSt has to be designed with movement of a BMI device and subject alertness level in mind.

We can pose a number of other questions based on our results which we hope will be answered using larger neuronal sampling and human experiments or behavioral tests in other primates. For instance, would the increase in information flow from the thalamic subnuclei to the cortex when microstimulating in the thalamus affect the way other sensory modalities are simultaneously perceived and processed in the cortex? Is it possible to amplify another information carried by nearby relay neurons, such as pain signals which have been shown to be caused by thalamic microstimulation (Lenz et al., 1995), only by the gross amplification of total information flow from the thalamus to the cortex? Or could we assume that specific communication occurs in labeled lines and everything else can be assumed to work more or less normally? Could the fact that thalamic modulators outnumber drivers (Sherman and Guilery, 2006) mean the VPL, other factors permitting, can be trusted to be the best place to MiSt for somatosensory neuroprosthesis (SSNP) as less drivers may mean less susceptibility to failure signal transmission? The above outstanding questions, especially those that are neuroprosthesis related, are the subject of our continued investigation.

## Author contributions

Mulugeta Semework conceived and designed the work, performed experiments, acquired and analyzed the data, and wrote the manuscript. Marcello DiStasio helped in data interpretation, edited, and critically reviewed the manuscript.

### Conflict of interest statement

The authors declare that the research was conducted in the absence of any commercial or financial relationships that could be construed as a potential conflict of interest.
